# Transforming growth factor-ß as a therapeutic target for the cardiac damage of Chagas disease

**DOI:** 10.1590/0074-02760210395

**Published:** 2022-02-25

**Authors:** Mariana Caldas Waghabi, Roberto Rodrigues Ferreira, Rayane da Silva Abreu, Wim Degrave, Elen Mello de Souza, Sabine Bailly, Jean-Jacques Feige, Tania C de Araújo-Jorge

**Affiliations:** 1 Fundação Oswaldo Cruz-Fiocruz, Instituto Oswaldo Cruz, Laboratório de Genômica Funcional e Bioinformática, Rio de Janeiro, RJ, Brasil; 2 Fundação Oswaldo Cruz-Fiocruz, Instituto Oswaldo Cruz, Laboratório de Virologia Molecular, Rio de Janeiro, RJ, Brasil; 3 Institut National de la Santé et de la Recherche Médicale, Biology of Cancer and Infection Laboratory, Grenoble, France; 4 Fundação Oswaldo Cruz-Fiocruz, Instituto Oswaldo Cruz, Laboratório de Inovações em Terapias, Ensino e Bioprodutos, Rio de Janeiro, RJ, Brasil

**Keywords:** transforming growth factor beta, therapeutic, target, cardiac, damage, Chagas disease

## Abstract

Transforming growth factor beta (TGF-β) is deeply involved on the pathogenesis of Chagas disease. Our group has been investigating the participation of this pleiotropic cytokine in different aspects of Chagas disease over the last 20 years. Important observations have been made, such as: (i) the ability of *Trypanosoma cruzi* in activating latent TGF-β; (ii) the potential involvement of TGF-β pathway on *T. cruzi* invasion of host cells; (iii) association of TGF-β with parasite intracellular replication; (iv) cardiac fibrosis development and maintenance; (v) disruption of Connexin-43 plaque structures and (vi) inflammation and immune response. In this perspective article we intend to discuss the advances of the potential use of new therapies targeting TGF-β to treat the cardiac alterations of Chagas disease-affected patients.

The parasite load followed by the inflammatory imbalance occurred during the acute phase leads to different possible scenarios during the chronic phase. Chronic chagasic cardiomyopathy (CCC) is a complex disease with many possible clinical manifestations, in which treatment becomes a challenge. Heart fibrosis is often observed on CCC, as a scar due to inflammatory response against the presence of the parasite.^1^ Chagas disease (CD) treatment is still directed toward parasite eradication with trypanocidal compounds, such as nifurtimox and benznidazole, and for disease manifestations observed mainly in the long-lasting chronic phase, in order to control thromboembolic events, heart arrhythmias, and reducing the risk of sudden death. Current options for the treatment of CCC are based on generic therapeutic strategies that do not differ from those in other cardiomyopathies: diuretics, beta-blockers, angiotensin-converting enzyme inhibitors, and spironolactone.^2^ To date, the use of drugs directed to contain and/or reverse cardiac fibrosis is not a practice in clinics.

Transforming growth factor beta (TGF-β) is a key cytokine in fibrotic events. TGF-β is a pleiotropic cytokine, secreted under a latent form by almost all types of cells and needs to be activated into its mature form to develop its biological functions. Active TGF-β then binds to its membrane receptors: TGF-β receptor-type I (TβRI/ALK5) and -type II (TβRII). Ligand binding stimulates the phosphorylation of intracellular proteins of the classical pathway, Smad2/3, and some alternative pathways Erk, JNK, p38, PI3K, inducing a broad range of cellular responses, including the regulation of more than 500 genes.^3^ Heart fibrosis was shown to be mediated by TGF-β, as it is involved on the production of the expression of several matrix components, inhibition of the secretion of matrix-degrading proteases and stimulation of the synthesis of protease inhibitors.^4^

Our group have first identified a correlation between higher circulating levels of TGF-β with worse prognosis of patients with the cardiac form of chronic Chagas disease.^1^ ^,^ ^5^ We first addressed the question: Does TGF-β circulating levels could be a predictor biomarker of CD outcome during the chronic phase? After 10 years, we retrospectively analysed the all-cause mortality of these patients and TGF-β1 was higher among patient who died than in survivors.^6^ This was observed in the group of patients with normal to mild LV systolic dysfunction but not in patients with advanced heart failure (HF). Thus, TGF-β1 would be considered as an important determinant of CD patients’ outcome at earliest stages of the disease.^6^

Given the central role of TGF-β in the myocardial fibrosis development in patients with CCC, an important question was raised: Which host factors would guide TGF-β release and/or activation after *Trypanosoma cruzi* infection? Would it be related to host genetic background? Many groups have been investigating the association of single nucleotide polymorphism (SNP) present in cytokine genes with the susceptibility to develop CD and/or to progress to the cardiac symptomatic form of chronic disease. Some important findings were described: SNP observed on IFN-γ, MIF, IL-4, TNF, TGF-β, and IL-18 were associated to the risk to develop Chagas disease, but no association was observed with disease progression.^7^ Calzada et al. first demonstrated that the TGFB1 polymorphism at codon 10 was associated to Chagas disease susceptibility in Colombian and Peruvian cohorts.^8^ Then, we developed a similar study in a Brazilian cohort and found a correlation between TGF-β1 SNP, present at position -509 and at the codon 10, with CD susceptibility.^9^ It was previously reported that elevated serum levels of TGF-β1 was associated to TGFB1 SNP: TGFB1 promoter -509 C>T polymorphism (rs1800469).^10^ We could then speculate that patients presenting SNPs in the TGFB1 gene, in case of *T. cruzi* infection, would release higher TGF-β levels, favoring parasite entry and replication inside cells and establishment of chronic disease. Thus, it should be further confirmed by investigating the presence of SNPs in the TGFB gene associated to measuring TGF-β levels during the acute phase of the human disease, in order to correlate its increase with Chagas disease outcome.

These set of observations correlating TGF-β with a worse prognosis of patients with CCC, lead this cytokine as an interesting target for therapies focusing on its blockage to impair the development of or reverse cardiac fibrosis. Specific anti-fibrotic therapies targeting TGF-β are under investigation all over the world and many clinical trials were applied. Using the terms fibrosis and TGF-β to search studies in the www.clinicaltrials.gov site, 68 results were retrieved in which Phase I/II clinical trials were mainly develop to pulmonary and liver fibrosis, and skin scars; few studies were developed for kidney, heart and cancer pathologies. These studies comprise the use of Anti- TGF-β compounds and/or inhibitors of TGF-β receptors: antisense nucleotides that block TGF-β mRNA (e.g., trabedersen), monoclonal antibodies that block TGF-β isoforms (e.g., lerdelimumab, metelimumab, fresolimumab), soluble receptor ectodomains that sequester TGF-β (P144), or small molecule inhibitors of the TGF-β receptor 1 and/or 2 (SB 431542, GW 788388, LY2157299). Although their potential benefits are evident, the clinical use of TGF-β pathway inhibitors should be deeply analysed as several TGF-β receptor kinase inhibitors appeared to induce heart valve lesions in preclinical models.^11^ Therefore, the search for safe drugs targeting TGF-β signaling are still required.

In the last years, our group developed in vivo experimental models of the CD acute and chronic phases with important reproducible clinical features. When inhibiting TGF-β pathway at early stages of the acute phase of CD, we observed reduced *T. cruzi* parasitaemia followed by reduced cardiac damage and fibrosis.^12^ ^,^ ^13^ The use of other compounds was also associated to the TGF-β role in maintenance of cardiac fibrosis, the anti-fibrotic effect observed by ganglioside GM1 administration was associated with a significant reduction in the myocardial expression of TGF-β.^14^ We then proposed to test if TGF-β inhibitors could also be active in a more complex scenario of CD. During the chronic phase of a pre-clinical model of CD, in which mice were treated after 120 dpi by oral administration with GW788388, a TGF-β receptor kinase inhibitor, important findings were observed: reduced levels of circulating TGF-β, lower phosphorylation of Smad 2/3 and reversion of deposition of ECM proteins in the heart, as well as improved heart rate and function with normal left ventricular ejection fraction.^15^ We further explored the possible mechanisms involved on these positive effects of TGF-β pathway blockage and found that the balance between MMPs and their tissue inhibitors, TIMPs, is pivotal in the remodeling of ECM deposition and reduction of fibrosis.^15^

It is known that the neonatal mammalian heart has regenerative potential, which is lost during the adult life. This feature brings complexity when cardiomyocytes are damaged after injury, as it could not be replaced, thus preventing heart regeneration. Nevertheless, new studies are showing the potential of adult mouse cardiomyocytes in reversible dedifferentiating on fetal-like cardiomyocytes thus making possible cardiac regeneration.^16^ ^,^ ^17^ After the positive observations of cardiac fibrosis reversion and improvement of heart function addressed by echocardiography analysis, after inhibiting TGF-β pathway, we intended to investigate if functional cardiac cells could be replaced on the fibrotic area. Our group showed cardiac recovery after GW788388 administration during the chronic phase of the experimental *T. cruzi* infection, with increased expression of GATA-6 and Tbox-5 mRNA and the presence of Sca-1+ cells, suggesting the emergence of cells with high potential of cardiac phenotype.^15^

These important findings highlight the possible use of TGF-β as an effective strategy and a therapeutic target to improve cardiac function with potential heart regeneration. Therefore, we hope to contribute to translate important pre-clinical knowledge on the TGF-β inhibition into Chagas disease clinical applications. To become a reality, it would need the participation and commitment of pharmaceutics industry worldwide in partnership with universities and research institutions to contribute with the development of safe compounds with affordable value to treat CD, a neglected tropical disease. The central role of TGF-β on the progression of Chagas disease in different aspects, supports the use of inhibitors of its pathway on chronic chagasic cardiomyopathy.
